# Amino acid sequence diversity of the major human papillomavirus capsid protein: Implications for current and next generation vaccines^☆^

**DOI:** 10.1016/j.meegid.2013.05.013

**Published:** 2013-08

**Authors:** Amina I. Ahmed, Sara L. Bissett, Simon Beddows

**Affiliations:** Virus Reference Department, Public Health England, London, UK

**Keywords:** Papillomavirus, HPV, Diversity, Entropy, L1

## Abstract

Despite the fidelity of host cell polymerases, the human papillomavirus (HPV) displays a degree of genomic polymorphism resulting in distinct genotypes and intra-type variants. The current HPV vaccines target the most prevalent genotypes associated with cervical cancer (HPV16/18) and genital warts (HPV6/11). Although these vaccines confer some measure of cross-protection, a multivalent HPV vaccine is in the pipeline that aims to broaden vaccine protection against other cervical cancer-associated genotypes including HPV31, HPV33, HPV45, HPV52 and HPV58. Both current and next generation vaccines comprise virus-like particles, based upon the major capsid protein, L1, and vaccine-induced, type-specific protection is likely mediated by neutralizing antibodies targeting L1 surface-exposed domains.

The aim of this study was to perform an *in silico* analysis of existing full length L1 sequences representing vaccine-relevant HPV genotypes in order to address the degree of naturally-occurring, intra-type polymorphisms. In total, 1281 sequences from the Americas, Africa, Asia and Europe were assembled. Intra-type entropy was low and/or limited to non-surface-exposed residues for HPV6, HPV11 and HPV52 suggesting a minimal effect on vaccine antibodies for these genotypes. For HPV16, intra-type entropy was high but the present analysis did not reveal any significant polymorphisms not previously identified. For HPV31, HPV33, HPV58, however, intra-type entropy was high, mostly mapped to surface-exposed domains and in some cases within known neutralizing antibody epitopes. For HPV18 and HPV45 there were too few sequences for a definitive analysis, but HPV45 displayed some degree of surface-exposed residue diversity. In most cases, the reference sequence for each genotype represented a minority variant and the consensus L1 sequences for HPV18, HPV31, HPV45 and HPV58 did not reflect the L1 sequence of the currently available HPV pseudoviruses. These data highlight a number of variant amino acid residues that warrant further investigation for vaccine and natural history studies of HPV.

## Highlights

• We evaluated amino acid diversity of the major capsid protein of HPV.
• Residues displaying high entropy were found within surface-exposed domains.
• We discuss the implications of this diversity on the current and next generation HPV vaccines.

## 1 Introduction

Cervical cancer is the third most common cancer of women, accounting for an estimated 530,000 cases and 275,000 deaths per annum worldwide (Arbyn et al., 2011). Although there are some geographical differences, human papillomavirus (HPV) genotypes HPV16 and HPV18 are associated with approximately 70% of cervical cancer cases (Li et al., 2010). Virus-like particle (VLP) vaccines (Cervarix® and Gardasil®) based on the major capsid (L1) proteins of HPV16 and HPV18 have demonstrated almost 100% efficacy against high grade lesions associated with these two types in clinical trials (Lu et al., 2011; Romanowski, 2011). Gardasil® also contains VLP representing HPV6 and HPV11 which are associated with the development of genital warts.

Serological assays for the evaluation of vaccine antibody responses and natural history studies of HPV infection include competitive and direct immunoassays (Dias et al., 2005; Giannini et al., 2006) and a pseudovirus-based neutralization assay (Pastrana et al., 2004). All these systems have potential shortcomings that affect their utility in monitoring vaccine-induced antibody responses (Schiller et al., 2012). However, the detection of high titer, type-specific neutralizing antibodies in the serum and cervico-vaginal secretions of human vaccines (Einstein et al., 2009) and antibody-mediated protection in animal models (Longet et al., 2011) has led to the reasonable assumption that antibodies are the principal mediators of type-specific vaccine-induced protection (Schiller and Lowy, 2012). Type-specific seroconversion following natural infection is less efficient (Carter et al., 2000) but appears to be associated with some degree of protection against subsequent infection (Lin et al., 2013).

Closely-related HPV types within the alpha-papillomavirus species groups A9 (HPV16-like: HPV31, HPV33, HPV35, HPV52 and HPV58) and A7 (HPV18 like: HPV39, HPV45, HPV59 and HPV68) are associated with a further *ca*. 25% of cervical cancers worldwide, although there are geographical differences in the relative distribution of these types (Li et al., 2010). The current generation of HPV vaccines afford some degree of cross-protection against a few of these types including HPV31, HPV33 and HPV45 but probably not HPV52 and HPV58 (Lu et al., 2011; Romanowski, 2011). This protection is at least coincident with the detection of neutralizing antibodies in the serum of vaccinated individuals (Draper et al., 2011; Einstein et al., 2011; Kemp et al., 2011). The limited degree of cross-protection afforded by the current generation of VLP-based vaccines has led to the development of a candidate next generation multivalent VLP-based vaccine to extend coverage to a wider array of HPV types (Van de Velde et al., 2012).

Despite the excellent proof-reading ability of host cell polymerases, the approximately 8 kb double-stranded DNA genome of HPV displays a certain degree of intra-type genome polymorphism (Chen et al., 2009, 2011). Such variants are predicted to have arisen over millennia due to the slow evolutionary rate of the genome (estimated to be 10^−8^ base substitutions per site per year) (Chen et al., 2009). The extent of HPV genome diversity, the interplay between positive and negative selection pressures and genetic drift, and the biological consequences of such variation are largely unclear (Bernard et al., 2006).

Serological cross-reactivity in ELISA (Cheng et al., 1995; Touze et al., 1998) and neutralization (Pastrana et al., 2001) assays using targets representing HPV16 variants suggested that the most common HPV16 L1 polymorphisms are unlikely to adversely influence the effectiveness of an HPV vaccine based upon a single HPV16 L1 sequence. This hypothesis appears to have been confirmed by HPV16/18 vaccine efficacy data reported from clinical trials carried out in the Americas, Europe and the Asia–Pacific region (Munoz et al., 2010; Paavonen et al., 2007).

VLP assembly follows a step-wise process in which five L1 monomers form an intermediate structure (known as a capsomer) and then these pentameric subunits associate to form an icosahedral structure of 72 capsomers (Chen et al., 2000). The highest degree of inter-type diversity is located within the external loops of the L1 protein and is thought to contribute to the differences in antigenicity between papillomavirus types (Bishop et al., 2007; Chen et al., 2000). Until recently, most studies examining intra-type variation of the major capsid protein have focussed on small fragments of the L1 gene. More recently, a number of studies have published full length L1 or full genome HPV sequences and consequently a detailed structural picture of intra-type variation is now possible. This study sought to perform an evaluation of worldwide L1 amino acid diversity for a range of vaccine-relevant HPV types, using available full length L1 sequences, and postulate upon the impact that such polymorphisms would have on current and candidate L1-based vaccines.

## 2 Materials and methods

### 2.1 Source of available sequences

Reference sequence accession numbers and the taxonomy identifiers for each HPV type are as indicated: HPV6 (X00203; txid31552; [6a] txid37122; [6b] txid10600), HPV11 (M14119; txid10580), HPV16 (HPV16R: corrected version of K02718 (Myers et al., 1995); txid333760), HPV18 (HPV18R: AY262282, corrected version of X05015; txid333761), HPV31 (J04353; txid10585), HPV33 (M12732; txid10586), HPV45 (X74479; txid10593), HPV52 (X74481; txid10618) and HPV58 (D90400; txid10598) (de Villiers et al., 2004). The reference sequences are the same as those given in the Papillomavirus Episteme database (http://pave.niaid.nih.gov). Sequences were downloaded from the National Center for Biotechnology Information (NCBI; http://www.ncbi.nlm.nih.gov/) between November and December 2012. A referenced source list of the sequences used in this study can be found in Supplementary Table S1. Only full length L1 sequences were considered for this analysis. For some studies, only one sequence was deposited in the NCBI database to represent multiple occurrences of a variant; in these cases, the prevalence of individual variant sequences was calculated using the numbers indicated in the relevant publication. The consensus sequence was generated from the panel of assembled sequences for each genotype by selecting the most frequent amino acid present at a given position, which may or may not be the same as the reference sequence.

### 2.2 Site-specific residue variation and mapping

Sequences were aligned, translated and exported as a FASTA formatted file using MEGA v4.1 (Tamura et al., 2007). Residue positions were numbered according to the initiating ATG codon of the L1 protein coding sequence (CDS; http://pave.niaid.nih.gov) of the appropriate reference sequence, according to convention (Bishop et al., 2007; Chen et al., 2000). For example, and for the purposes of clarity, the HPV16 L1 protein starts with the MSLW motif. Amino acid substitutions are designated X123Y, where X is the amino acid at reference sequence residue position 123 that is substituted by amino acid Y in the variant. Deletions are indicated by a Δ. Site-specific Shannon entropy was determined using the aligned amino acid sequences for each type using the *Entropy One* program on the Los Alamos National Laboratory website (http://www.hiv.lanl.gov/content/sequence/ENTROPY/entropy_one.html) (Korber et al., 1994). For comparison purposes, a level of 5% residue variation equates to an entropy score of *ca*. 0.18. Protein secondary structures (amino [Nt] and carboxy [Ct] termini, α-helices, β-sheets and external loops [BC, DE, EF, FG, HI]) were highlighted accordingly (Bishop et al., 2007). Variant residues were mapped to the surface of the HPV16 capsomer crystal structure (PDB code: 2R5H) using Swiss-PDP viewer v4.0 (Deep View) (Guex and Peitsch, 1997).

### 2.3 Statistical analysis

Differences in entropy between loop and non-loop residues (non parametric, Mann–Whitney *U* test) and differences in proportions in geographic distribution (2-tailed Fisher’s exact test) were evaluated using Stata 12.0 (StataCorp, Tx., USA).

## 3 Results and discussion

### 3.1 HPV6/11

**Fig. 1:**
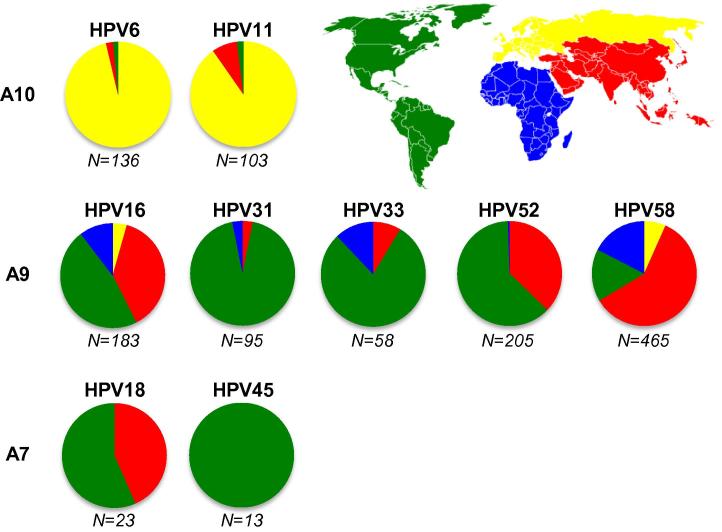
Geographical distribution of HPV full length L1 sequences. Total numbers of available full length L1 sequences for each HPV type are indicated beneath each chart. Alpha-papillomavirus species groups are as indicated: A10 (HPV6, HPV11), A9 (HPV16, HPV31, HPV33, HPV52 and HPV58) and A7 (HPV18, HPV45). The regional proportion of L1 sequences are colored according to the inset map: Americas (green), Europe (yellow), Africa (blue) and Asia (red).

HPV6 and HPV11 genotypes were represented by *n* = 136 and *n* = 103 sequences, respectively, with the majority (94%) being from Europe (Fig. 1). There were no full length sequences from Africa and few from the Americas (2%) or Asia (4%). The reference sequences for HPV6 and HPV11 were the same as their respective consensus sequence and represented 76% (95% CI, 68–83%) and 86% (95% CI, 78–92%) of HPV6 and HPV11 full length L1 sequences, respectively. In both cases, the pseudovirus L1 sequence was the same as the consensus and the reference sequence.

**Fig. 2:**
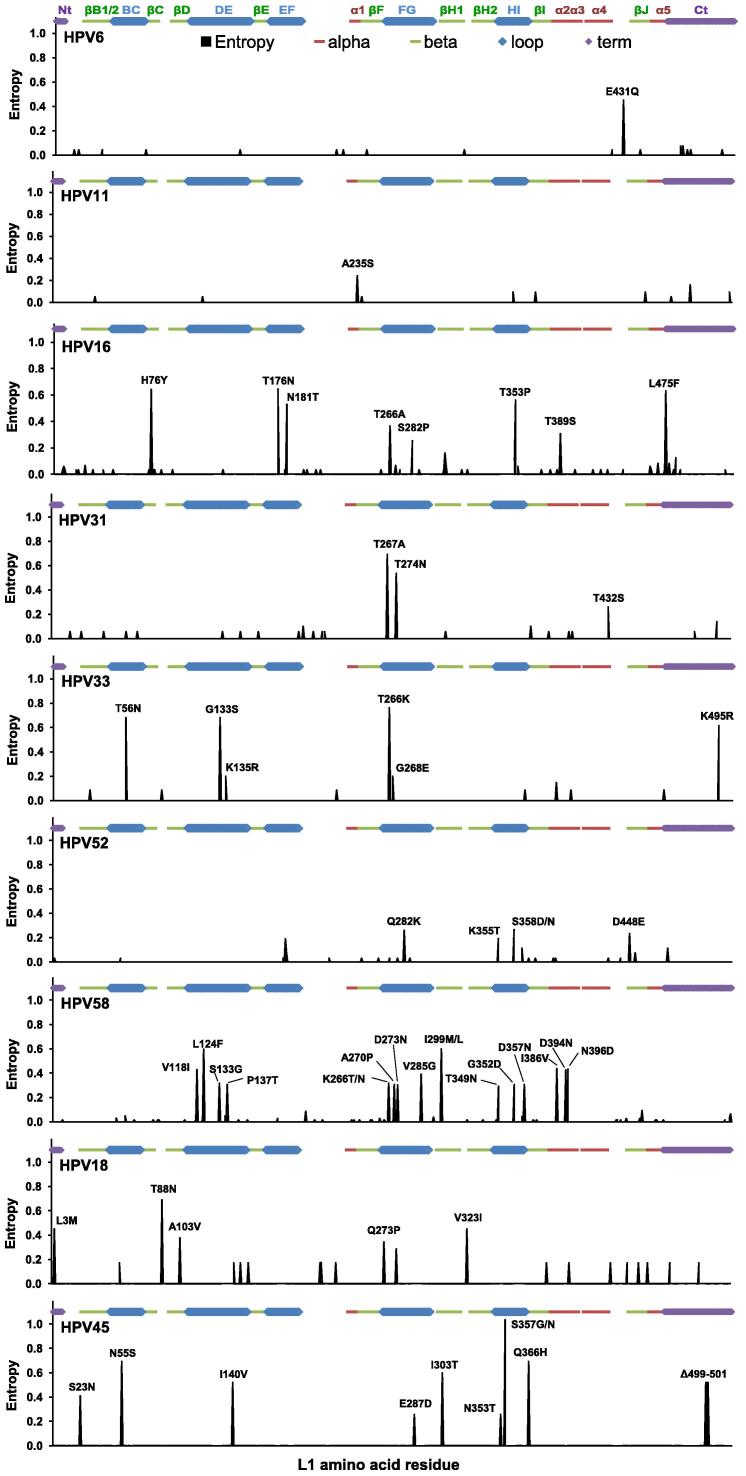
Intra-type, site-specific amino acid residue entropy. Residue variation estimated using Shannon entropy, wherein a value of zero reflects site-specific conservation and higher values indicate increasing degrees of site-specific variation. A level of 5% residue variation equates to an entropy score of *ca*. 0.18. Site-specific variation(s) above this level are indicated by convention, numbered according to the reference sequence for that HPV type. The positions of major structural elements are indicated.

HPV6 L1 sequence variation was low (Fig. 2) with 17% of sequences incorporating an E431Q substitution in the α4-βJ region (Table 1). The E431Q substitution does not appear to have an impact on VLP stability (Caparros-Wanderley et al., 1999). HPV6 vaccine antibodies are evaluated by competition with the type-specific neutralizing monoclonal antibody (MAb) H6.M48 (Dias et al., 2005; Smith et al., 2008). The epitope of this MAb appears to localize to the BC loop, with possible contribution from the EF loop, while antibodies generated during natural infection appear to bind to a range of epitopes (McClements et al., 2001; Orozco et al., 2005).

HPV11 L1 sequence variation was low (Fig. 2) with 7% of sequences incorporating an A235S substitution in the α1 region (Table 1). HPV11 vaccine antibodies are monitored through competition with the type-specific neutralizing MAb K11.B2 (Dias et al., 2005; Smith et al., 2008). The epitope of this MAb is unclear. MAb K11.B2 replaced MAb H11.B2 during development of the competitive assay and the epitope of this latter MAb was identified within the DE loop (Ludmerer et al., 1996; Orozco et al., 2005). Antibodies generated following natural infection appear to target epitopes in the C-terminal (Ct) portion downstream of the DE loop (Wang et al., 2003).

There were too few non-European sequences available to evaluate the geographical distribution of the HPV6 E431Q and HPV11 A235S variants.

### 3.2 HPV16, HPV31, HPV33, HPV52 and HPV58

**Fig. 3:**
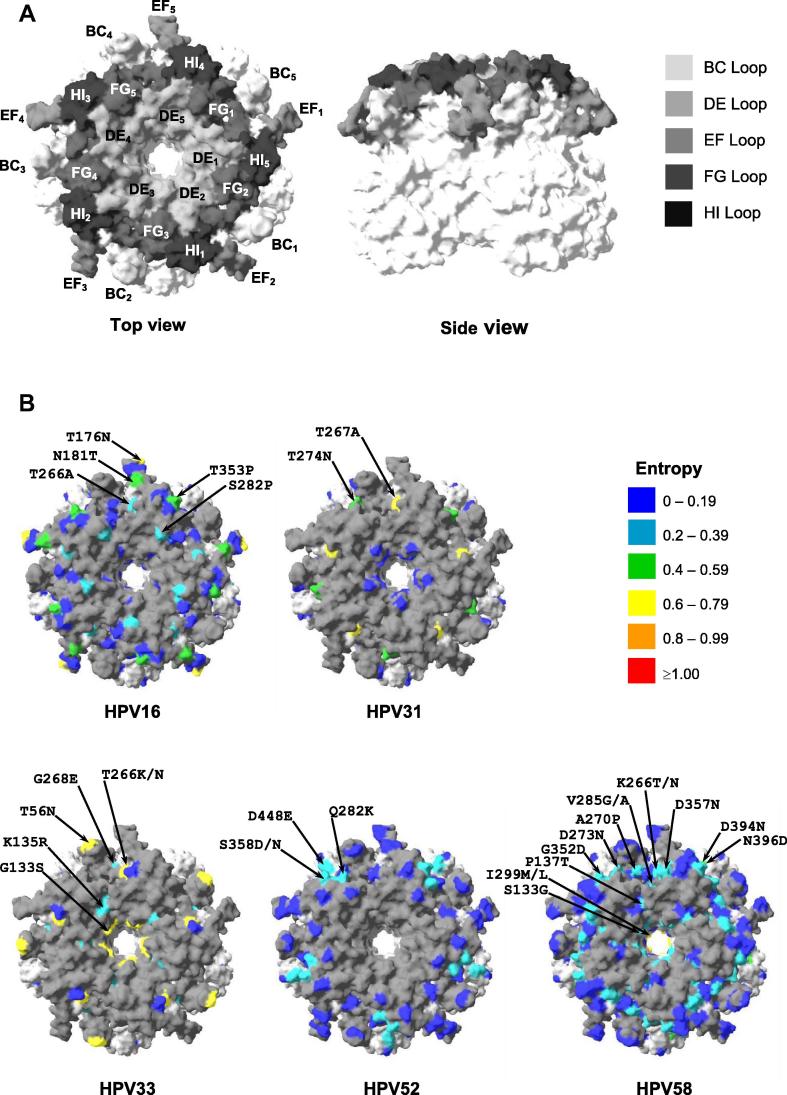
Crystal model surface representation of variant amino acid residues. (A) top view (left panel) and side view (right panel) of HPV16 pentamer (PDB code: 2R5H) with variable loops shaded as indicated. (B) Shannon entropy scores for indicated A9 HPV types are mapped onto top view of HPV16 pentamer structure according to the indicated scheme. Only those variant amino acid residues located on the surface of the capsomer are shown. While all five monomers of the capsomer are pictured, and all appropriate sites of variation are highlighted, only one of the five copies of a residue are indicated with the amino acid mutation (black arrows), and all the variations for a given HPV type may not be indicated on the same monomer.

HPV16 (*n* = 183), HPV52 (*n* = 205) and HPV58 (*n* = 465) were well represented within the dataset, with HPV31 (*n* = 95) and HPV33 (*n* = 58) less so. Each genotype was represented by sequences from Africa, the Americas and Asia, albeit to varying degrees, but European sequences were only available for HPV16 and HPV58, at 3% and 7% of the total, respectively (Fig. 1). The available number of sequences and the degree of intra-type variation was sufficient to enable mapping the entropy score of surface exposed residues for each A9 genotype onto the crystal structure of the HPV16 capsomer (Fig. 3).

The reference sequence for HPV16 represented only 7% (95% CI, 3–11%) of sequences. The consensus sequence differed from the reference by a T266A substitution in the FG loop, represented 37% (95% CI, 30–44%) of sequences overall and could be found in all four geographical regions. The HPV16 pseudovirus L1 sequence was identical to the consensus. In addition to the T266A polymorphism, several variable sites within structural domains (H76Y, T389S, L475F) or within the EF (T176N, N181T), FG (S282P) and HI (T353P) loops were found in these sequences (Fig. 2), in a variety of combinations (Supplementary Fig. S1). Surface-exposed residues that exhibited the highest degree of variation did not appear to form a distinct cluster but instead could be found dispersed along the ridge of the EF loop and its base (adjacent to the FG/HI loops) (Fig. 3A, 3B HPV16). The H76Y, T176N, S282P and L475F mutations were overrepresented in sequences from Africa, while the H76Y and L475F mutations were underrepresented in sequences from Asia relative to the overall mean (Table 1). The HPV16 VLP in the Cervarix® vaccine matches the consensus sequence, including the T266A substitution (Deschuyteneer et al., 2010). HPV16 vaccine antibodies are monitored by competition with the type-specific neutralizing MAb H16.V5 (Dias et al., 2005; Giannini et al., 2006; Smith et al., 2008). This MAb targets a conformational epitope in the FG loop, with possible contribution from the HI loop (Carter et al., 2003) and is thought to represent a significant proportion, but not the entirety, of antibody specificities produced during natural infection (Carter et al., 2003; Wang et al., 1997). Using a series of HPV16:HPV31 loop-swap hybrid VLP, HPV16-specific antibodies generated during natural infection appeared to target the DE, FG, and HI loops (Carter et al., 2006). The H76Y, T176N, N181T, S282P, T353P, T389S and L475F mutations have been substituted into an HPV16 pseudovirus L1 backbone based upon the European variant 114/K (Accession no. EU118173, identical to the consensus reported here) and found not to have a significant impact on recognition by antibodies elicited to HPV16 (Pastrana et al., 2001). The binding and neutralization of MAb H16.E70, however, appears to be affected by substitutions in the FG loop, including the reciprocal A266T mutation which was present in *ca*. 11% of sequences (Carter et al., 2003; Roden et al., 1997; Varsani et al., 2006).

The reference sequence for HPV31 represented only 12% (95% CI, 6–20%) of sequences and was the same as the pseudovirus sequence. The HPV31 L1 protein exhibited variation in the α4-βJ region (T432S in 7% of sequences) and the FG loop (T267A and T274N; in 49% and 78% of sequences, respectively) (Fig. 2 and Table 1), in a limited number of combinations (Supplementary Fig. S1). The T267A and T274N residues are located at distinct sites on the surface of the FG loop (Fig. 3A, 3B HPV31). There were too few African and Asian sequences to ascertain the proportions of their distribution outside of the Americas. HPV31 MAbs have been shown to target both the EF and FG loops. MAb H31.A6 bound VLP containing the HPV31 EF loop in a HPV16 backbone, but not the reciprocal mutant (Carter et al., 2003). Three HPV31 MAbs have been identified as targeting the FG loop, including an epitope encompassing the T267A variant residue (Fleury et al., 2009), while another study demonstrated that cross-reactive HPV16/HPV31 MAbs share a common binding motif in the FG loop (Carpentier et al., 2005). The FG loop variants T267A and T274N may, therefore, have an impact on the epitope configuration of some HPV31-specific antibodies.

The reference sequence for HPV33 represented 48% (95% CI, 35–62%) of sequences examined and was identical to both the consensus and pseudovirus sequences. There were six sites of significant variation in the HPV33 L1 protein, including variation in the BC (T56N), DE (G133S, K135R), FG (T266K/N, G268E) loops and the Ct (K495R) (Fig. 2, Table 1). Despite the number of individual variant residues, the number of variant sequences containing multiple variant sites was fairly limited (Supplementary Fig. S1). The major variant residues of HPV33 were dispersed across the surface of the capsomer (Fig. 3A and B HPV33), including the tip of the BC loop, a small cleft at the base of the BC and EF loops (adjacent to the FG loop) and the capsomer lumen. The most common variant positions (T56N, G133S, T266K, K495R) were found in the Americas, Asia and Africa, although the latter two regions were represented by too few sequences (*n* = 5 and 7, respectively) to evaluate their proportionate distribution. Three HPV33 MAbs (H33.J3, H33.B6 and H33.E12) have been identified as binding conformational epitopes within the BC, DE and FG loops (Roth et al., 2006). H33.J3 appears to target a discrete epitope within the BC loop of HPV33 and was able to neutralize an HPV16:HPV31BC hybrid and wild-type HPV33 but not wild-type HPV16 (Roth et al., 2006). Naturally occurring residue variations within the BC, DE and FG loops, as highlighted in this study, may therefore affect recognition by these MAbs and, by implication, neutralizing antibodies generated to the HPV33 L1 protein.

The reference, consensus and pseudovirus sequences for HPV52 were identical, representing 81% (95% CI, 75–87%) of sequences and could be found in the Americas, Asia and Africa. There were four main sites of variation in the HPV52 L1 protein (Fig. 2), located in the FG (Q282K) and HI (K355T, S358D/N) loops and the α4-βJ region (D448E) found on a limited number of variant sequences (Supplementary Fig. S1). Three of these (Q282K, K355T, S358D/N) appeared to form a distinct cluster along the edges of the FG/HI loops (Fig. 3A, 3B HPV52). Overall, these variants were present in <10% of sequences (Table 1) and tended to be underrepresented in sequences from Asia. There were few data available on the specificity of HPV52 MAbs (Rizk et al., 2008). HPV52 VLP incorporating the BC loop of HPV16 reduced, but did not eliminate, the binding of four HPV52 MAbs, suggesting that this region may contribute to the formation of an immunodominant domain (Carter et al., 2003). The implications of variation within the FG and HI loops, as outlined herein, are uncertain. The Q282K substitution restores a Lysine residue that, for HPV16 (K278) at least, is thought to be important for cell binding via heparin moieties (Knappe et al., 2007). No other HPV types exhibited significant (⩾5%) variation within these putative heparin-binding sites.

The reference sequence for HPV58 represented only 6% (95% CI, 4–9%) of sequences. The consensus sequence differed from the reference by L124F (DE loop) and I299M/L (βG1 region) substitutions, represented 62% (95% CI, 58–67%) of sequences examined and could be found in all four geographical regions. The pseudovirus sequence for HPV58 was the same as the reference and therefore did not represent the consensus of circulating sequences. Variation across the L1 protein was greater for HPV58 than any of the types evaluated (Fig. 2) and predominantly associated with the external loops (*p *< 0.001). Variant residues were found in internal domains including βG1 (I299M/L), α2 (I386V), α2-α3 (D394N), α3 (N396D) and the DE (V118I, L124F, S133G, P137T), FG (K266T/N, A270P, D273 N, V285G/A) and HI (T349 N, G352D, D357N) loops. These variant residues could be found in various combinations within 21 distinct variant sequences (Supplementary Fig. S1). Most of the surface-exposed variant residues formed part of distinct clusters of sites between the bases of the BC and EF loops and the distal portion of the FG and HI loops (Fig. 3A and B HPV58). The pentamer lumen also exhibited a degree of variation. With the exceptions of the L124F, I299M/L and T349N mutations the remainder were highly overrepresented in African and underrepresented in Asian sequences (Table 1). A limited number of HPV58 MAbs have been generated and these appear to exhibit a range of target specificities (Brendle et al., 2010) although detailed epitopes have not been defined.

### 3.3 HPV18 and HPV45

There were few HPV18 (*n* = 23) and HPV45 (*n* = 13) full length L1 sequences available for analysis. The majority of the HPV18 and all of the HPV45 sequences were from the Americas, with no available sequences from Europe or Africa (Fig. 1), prohibiting an evaluation of geographic distribution of individual variants.

The reference sequence for HPV18 represented 39% (95% CI, 20–61%) of sequences. The consensus sequence for HPV18 differed from the reference by a T88N substitution (present in 57% of sequences) in the βC-βD region, although the consensus sequence itself was only represented by a single sequence. The pseudovirus sequence for HPV18 was the same as the reference and therefore did not represent the consensus sequence. The majority of substitutions were located within internal structural domains (L3M, A103V, V323I) (Fig. 2), with the exception of one substitution, Q273P, found within the FG loop (Table 1). These variant residues were found in a limited number of combinations (Supplementary Fig. S1). HPV18 VLP used in the Cervarix® vaccine appear to contain the T88N, A103V and Q273P but not the L3M and V323I substitutions (Deschuyteneer et al., 2010). HPV18 vaccine antibodies are monitored by competition with MAb H18.J4 (Dias et al., 2005; Giannini et al., 2006; Smith et al., 2008) which recognizes a predominantly type-specific conformational neutralizing epitope (Smith et al., 2007).

The reference sequence for HPV45 represented only 1/13 sequences (8%; 95% CI, 2–36%). There were a number of variant residues relative to the reference sequence (Fig. 2) including those in the structural domains Nt-βB1 (S23N), βG1 (I303T), HI-β1 (Q366H), Ct (Δ499–501) and the BC (N55S), DE (I140V), FG (E287D) and HI (N353T, S357G/N) loops but too few sequences overall to evaluate the proportions of these variants appropriately (Table 1). These variant residues were found in a limited number of combinations (Supplementary Fig. S1). The pseudovirus sequence for HPV45 was the same as the reference. MAbs raised against HPV45 VLP are directed towards conformational epitopes and can be either type-specific or cross-reactive (Smith et al., 2007), though the exact epitope(s) are not available.

## 4 Conclusions

The number of available full length L1 sequences for some of the HPV types limited the precision of estimates around the proportions of site-specific variation. Most A10 species group sequences were from Europe while the majority of the A7 and A9 species group sequences were from the Americas or Asia. There was a paucity of African sequences overall and of European sequences representing A7 and A9 species group genotypes. In addition, it is possible that the deposition of sequences has been skewed towards the reporting of more variant sequences rather than sequences that match the reference. Taken together, any empirical evaluation of site-specific residue variation will likely be somewhat biased by the disparate distribution of the available sequences, a common problem with this kind of assessment. Nevertheless, the number of sequences was sufficient to highlight the representativeness, or not, of the reference and pseudovirus sequences and significant sites of intra-type variation for further study.

The reference sequence was the same as the consensus sequence for HPV6, HPV11, HPV33 and HPV52 but for the other types represented a minority variant. The pseudovirus L1 sequence was representative of the consensus sequence for HPV6, HPV11, HPV16, HPV33 and HPV52. For the remainder (HPV18, HPV31, HPV45 and HPV58) the pseudovirus sequence was based upon the reference sequence and was therefore not representative of the majority of circulating HPV sequences. The lack of representation of the consensus sequences in the pseudoviruses may not be a significant issue provided naturally occurring deviations do not negatively impact the functional utility of the pseudovirus assay. For studies of natural infection, therefore, it may be appropriate to evaluate empirically the implications of these deviations from the pseudovirus sequence.

Inter-type entropy, generated using the consensus for each type, was high and localized to the external loops (*p* < 0.001; Supplementary Fig. S2) as expected (Chen et al., 2000). A difference in the intra-type entropy scores between the external loops and internal structural regions was only found for HPV58, however, reflecting both the high volume of available L1 sequences and the degree of variation for this HPV type. Intra-type entropy was more sporadic and generally of lower magnitude than similar evaluations for RNA viruses such as HIV-1 (Rhee et al., 2008; Woo et al., 2010), Influenza (Huang et al., 2012) and Norovirus (Jin et al., 2011) reflecting the excellent proof-reading capability of host cell polymerases and the apparent timescale in which these variants have arisen (Chen et al., 2009).

Sites of variation for HPV6, HPV11, and to some extent for HPV52, were rare or confined to a limited number of internal residues suggesting that the impact of these polymorphisms on recognition by L1-specific antibodies is unlikely to be significant. For HPV16, the most common sites of variation (apart from the reciprocal A266T variant) have already been evaluated (Pastrana et al., 2001) and demonstrate a limited effect on antibody recognition. For the remaining HPV types, the degree of site-specific variation was high and in some cases within domains implicated in the recognition by L1-specific antibodies. This was particularly evident for HPV31, HPV33 and HPV58. For HPV31 and HPV33 the residues exhibiting the highest degree of variation tended to be in discrete sites while for HPV58 these residues appeared to be localized into distinct clusters. Some HPV types exhibited a degree of variation within the lumen of the capsomer which has potential implications for the interaction with the minor capsid protein, L2 (Buck et al., 2008).

The consequences of these polymorphisms for recognition by antibodies elicited following natural infection or vaccination by the current and next generation vaccines warrants further investigation.

## Funding

This work received no specific grant from any funding agency in the public or commercial sectors.

## Figures and Tables

**Table 1:** Summary of L1 amino acid polymorphisms.

HPV	Residue	Region^a^	Geographical region^b^				Total	
			Americas	Europe	Africa	Asia	n/N	% (95%CI)
6	E431Q	α4-βJ	0/2	23/131		0/3	23/136	17 (11–24)
11	A235S	α1	0/2	7/93		0/8	7/103	7 (3–14)
								
16	H76Y	βC	35/86	0/8	**16/19**⁎	**12/70**⁎	63/183	34 (28–42)
	T176N	EF	35/86	0/8	**16/19**⁎	13/70	64/183	35 (28–43)
	N181T	EF	17/86	0/8	6/19	18/70	41/183	22 (17–29)
	T266A	FG	68/86	8/8	16/19	70/70	162/183	89 (83–93)
	S282P	FG	4/86	0/8	**7/19**⁎⁎	2/70	13/183	7 (4–12)
	T353P	HI	26/86	0/8	9/19	11/70	46/183	25 (19–32)
	T389S	α2	10/86	0/8	2/19	5/70	17/183	9 (6–14)
	L475F	Ct	34/86	0/8	**16/19**⁎	**10/70**⁎	60/183	33 (26–40)
31	T267A	FG	46/89		1/3	0/3	47/95	49 (39–60)
	T274N	FG	71/89		2/3	1/3	74/95	78 (68–86)
	T432S	α4-βJ	7/89		0/3	0/3	7/95	7 (3–15)
33	T56N	BC	18/46		3/7	4/5	25/58	43 (30–57)
	G133S	DE	18/46		3/7	4/5	25/58	43 (30–57)
	K135R	DE	2/46		0/7	1/5	3/58	5 (1–14)
	T266K/N	FG	20/46		3/7	5/5	28/58	48 (35–62)
	G268E	FG	2/46		0/7	1/5	3/58	5 (1–14)
	K495R	Ct	14/46		3/7	1/5	18/58	31 (20–45)
52	Q282K	FG	15/127		0/1	**0/76**⁎	15/205	7 (4–12)
	K355T	HI	10/127		0/1	0/76	10/205	5 (2–9)
	S358D/N	HI	14/127		0/1	**0/76**⁎	14/205	7 (4–11)
	D448E	α4-βJ	13/127		0/1	**0/76**⁎	13/205	6 (3–11)
58	V118I	DE	15/75	2/31	**46/81**⁎⁎⁎	**9/278**⁎⁎⁎	72/465	15 (12–19)
	L124F	DE	61/75	29/31	**38/81**⁎	205/278	333/465	72 (67–76)
	S133G	DE	8/75	1/31	**32/81**⁎⁎⁎	**4/278**⁎⁎⁎	45/465	10 (7–13)
	P137T	DE	8/75	1/31	**30/81**⁎⁎⁎	**4/278**⁎⁎⁎	43/465	9 (7–12)
	K266T/N	FG	8/75	1/31	**30/81**⁎⁎⁎	**4/278**⁎⁎⁎	43/465	9 (7–12)
	A270P	FG	8/75	1/31	**30/81**⁎⁎⁎	**4/278**⁎⁎⁎	43/465	9 (7–12)
	D273N	FG	8/75	1/31	**30/81**⁎⁎⁎	**3/278**⁎⁎⁎	42/465	9 (7–12)
	V285G/A	FG	9/75	1/31	**34/81**⁎⁎⁎	**7/278**⁎⁎⁎	51/465	11 (8–14)
	I299M/L	βG1	62/75	28/31	**38/81**⁎	211/278	339/465	73 (69–77)
	T349N	HI	1/75⁎	2/31	11/81	26/278	40/465	9 (6–12)
	G352D	HI	8/75	1/31	**30/81**⁎⁎⁎	**4/278**⁎⁎⁎	43/465	9 (7–12)
	D357N	HI	8/75	1/31	**30/81**⁎⁎⁎	**4/278**⁎⁎⁎	43/465	9 (7–12)
	I386V	α2	10/75	2/31	**46/81**⁎⁎⁎	**16/278**⁎⁎⁎	74/465	16 (13–20)
	D394N	α2-α3	14/75	2/31	**45/81**⁎⁎⁎	**10/278**⁎⁎⁎	71/465	15 (12–19)
	N396D	α3	15/75	2/31	**46/81**⁎⁎⁎	**10/278**⁎⁎⁎	73/465	16 (13–19)
								
18	L3M	Nt	3/13			1/10	4/23	17 (5–39)
	T88N	βC-βD	10/13			3/10	13/23	57 (34–77)
	A103V	βD	2/13			1/10	3/23	13 (3–34)
	Q273P	FG	2/13			0/10	2/23	9 (1–28)
	V323I	βG2-βH1	4/13			0/10	4/23	17 (5–39)
45	S23N	Nt-βB1	12/13				12/13	92 (64–100)
	N55S	BC	7/13				7/13	54 (25–81)
	I140V	DE	3/13				3/13	23 (5–54)
	E287D	FG	1/13				1/13	8 (0–36)
	I303T	βG1	4/13				4/13	31 (9–61)
	N353T	HI	1/13				1/13	8 (0–36)
	S357G/N	HI	11/13				11/13	85 (55–98)
	Q366H	HI-βI	7/13				7/13	54 (25–81)
	ΔT499	Ct	11/13				11/13	85 (55–98)
	ΔA500	Ct	11/13				11/13	85 (55–98)
	ΔS501	Ct	11/13				11/13	85 (55–98)

a L1 region incorporating substitutions or deletions (Δ) within amino (Nt) and carboxy (Ct) terminii, external loops (BC, DE, EF, FG, HI), alpha helices (α) or beta sheets (β).

b Proportion of regional sequences harboring indicated mutation relative to total number of sequences with differences highlighted in bold type.

⁎ *p*<0.05; (2-tailed Fisher’s exact test).

⁎⁎ *p*<0.01; (2-tailed Fisher’s exact test).

⁎⁎⁎ *p*<0.001 (2-tailed Fisher’s exact test).

## Appendix A Supplementary data

Supplementary Fig. S1 Coincidence of major variant residues for genotypes within the A9 and A7 species groups.Amino acid residue numbering and reference sequence shown for each genotype with percentage occurence of each variant in the dataset indicated below. For clarity, only those residue positions that vary individually at or above the 5% diversity threshold are shown. [N], number in parentheses highlight the total number of sequences included for indicated HPV genotype.

Supplementary Fig. S2 Inter-type, site-specific amino acid residue entropy. Residue variation estimated using Shannon entropy, wherein a value of zero reflects site-specific conservation and higher values indicate increasing degrees of site-specific variation. The positions of major structural elements are indicated.

Supplementary Table S1 Accession numbers and source references.
