# Supplementary material for: Amino acid sequence diversity of the major human papillomavirus capsid protein: Implications for current and next generation vaccines
Source: Infect Genet Evol. 2013 Aug;18:151–9. doi: 10.1016/j.meegid.2013.05.013 (PMC3769806; doi:10.1016/j.meegid.2013.05.013)
Supplement: Supplementary Fig. S1 — Coincidence of major variant residues for genotypes within the A9 and A7 species groups.Amino acid residue numbering and reference sequence shown for each genotype with percentage occurence of each variant in the dataset indicated below. For clarity, only those residue positions that vary individually at or above the 5% diversity threshold are shown. [N], number in parentheses highlight the total number of sequences included for indicated HPV genotype. [file mmc1.pdf]

**HPV16**

| 76 | 176 | 181 | 266 | 282 | 353 | 389 | 475 | |
|---|---|---|---|---|---|---|---|---|
| **H** | **T** | **N** | **T** | **S** | **T** | **T** | **L** | [183] |
| . | . | . | A | . | . | . | . | 49% |
| . | . | . | . | . | . | . | . | 10% |
| Y | N | T | A | . | P | S | F | 9% |
| Y | N | . | A | . | . | . | F | 8% |
| Y | N | . | A | . | P | . | F | 6% |
| . | . | T | A | . | . | . | . | 5% |
| Y | N | T | A | P | P | . | F | 4% |
| Y | N | . | A | P | P | . | F | 3% |
| Y | N | T | A | . | P | . | F | 2% |
| Y | N | T | A | . | . | . | . | 1% |
| . | N | . | A | . | P | S | . | 0.5% |
| Y | N | . | . | . | . | . | F | 0.5% |
| Y | N | T | . | P | P | . | F | 0.5% |
| Y | N | T | A | . | P | . | . | 0.5% |

**HPV31**

| 267 | 274 | 332 | |
|---|---|---|---|
| **T** | **T** | **T** | [95] |
| A | N | . | 49% |
| . | N | . | 28% |
| . | . | . | 15% |
| . | . | S | 7% |

**HPV33**

| 56 | 133 | 135 | 266 | 268 | 495 | |
|---|---|---|---|---|---|---|
| **T** | **G** | **K** | **T** | **G** | **K** | [58] |
| . | . | . | . | . | . | 52% |
| N | S | . | K | . | R | 29% |
| N | S | . | K | . | . | 12% |
| . | . | R | K | E | . | 5% |
| N | S | . | N | . | R | 2% |

**HPV52**

| 282 | 355 | 358 | 448 | |
|---|---|---|---|---|
| **Q** | **K** | **S** | **D** | [205] |
| . | . | . | . | 92% |
| K | T | D | E | 4% |
| K | . | D | E | 2% |
| K | . | . | . | 1% |
| . | . | N | . | 0.5% |
| . | T | . | . | 0.5% |

**HPV58**

| 118 | 124 | 133 | 137 | 266 | 270 | 273 | 285 | 299 | 349 | 352 | 357 | 386 | 394 | 396 | |
|---|---|---|---|---|---|---|---|---|---|---|---|---|---|---|---|
| **V** | **L** | **S** | **P** | **K** | **A** | **D** | **V** | **I** | **T** | **G** | **D** | **I** | **D** | **N** | [465] |
| . | F | . | . | . | . | . | . | M | . | . | . | . | . | . | 68% |
| . | . | . | . | . | . | . | . | . | . | . | . | . | . | . | 9% |
| I | . | G | T | T | P | N | G | . | . | D | N | V | N | D | 9% |
| . | . | . | . | . | . | . | . | . | N | . | . | . | . | . | 5% |
| I | . | . | . | . | . | . | . | . | N | . | . | V | N | D | 2% |
| . | F | . | . | . | . | . | . | M | . | . | . | V | . | . | 1% |
| I | . | . | . | . | . | . | . | . | . | . | . | . | N | D | 1% |
| I | . | . | . | . | . | . | A | M | N | . | . | V | N | D | 1% |
| I | F | . | . | . | . | . | . | M | . | . | . | V | N | D | 1% |
| . | . | . | . | . | . | . | . | M | . | . | . | . | . | . | 1% |
| . | F | . | . | . | . | . | . | . | N | . | . | . | . | . | 1% |
| I | . | . | . | . | . | . | . | . | . | . | . | V | N | D | 0.4% |
| I | . | . | . | . | . | . | A | . | . | . | . | V | N | D | 0.4% |
| . | F | . | . | . | . | . | . | L | . | . | . | . | . | . | 0.2% |
| . | F | . | . | . | . | . | . | M | . | . | . | V | N | D | 0.2% |
| . | F | G | . | . | . | . | . | M | . | . | . | . | . | . | 0.2% |
| I | . | . | . | . | . | . | A | M | N | . | . | V | . | D | 0.2% |
| I | . | G | T | N | P | N | G | . | . | D | N | V | N | D | 0.2% |
| I | . | G | T | T | P | . | G | . | . | D | N | V | N | D | 0.2% |
| I | . | G | T | T | P | N | G | . | . | D | N | V | . | D | 0.2% |
| I | F | G | . | . | . | . | . | M | . | . | . | V | N | D | 0.2% |

**HPV18**

| 3 | 88 | 103 | 273 | 323 | |
|---|---|---|---|---|---|
| **L** | **T** | **A** | **Q** | **V** | [23] |
| . | . | . | . | . | 43% |
| . | N | . | . | I | 17% |
| M | N | . | . | . | 17% |
| . | N | . | . | . | 9% |
| . | N | V | P | . | 9% |
| . | N | V | . | . | 4% |

**HPV45**

| 23 | 55 | 140 | 287 | 303 | 353 | 357 | 366 | 499 | 500 | 501 | |
|---|---|---|---|---|---|---|---|---|---|---|---|
| **S** | **N** | **I** | **E** | **I** | **N** | **S** | **Q** | **T** | **A** | **S** | [13] |
| N | . | . | . | . | . | G | . | - | - | - | 23% |
| N | S | . | . | T | . | N | H | - | - | - | 23% |
| N | S | V | . | . | . | G | H | - | - | - | 23% |
| . | . | . | . | . | . | . | . | . | . | . | 8% |
| N | . | . | . | . | . | . | . | . | . | . | 8% |
| N | . | . | D | . | . | G | . | - | - | - | 8% |
| N | S | . | . | T | T | N | H | - | - | - | 8% |

**Supplementary Figure S1. Coincidence of major variant residues for genotypes within the A9 and A7 species groups.**
Amino acid residue numbering and reference sequence shown for each genotype with percentage occurance of each variant in the dataset indicated below. For clarity, only those residue positions that vary individually at or above the 5% diversity threshold are shown. [N], number in parentheses highlight the total number of sequences included for indicated HPV genotype.
