# Supplementary material for: Amino acid sequence diversity of the major human papillomavirus capsid protein: Implications for current and next generation vaccines
Source: Infect Genet Evol. 2013 Aug;18:151–9. doi: 10.1016/j.meegid.2013.05.013 (PMC3769806; doi:10.1016/j.meegid.2013.05.013)
Supplement: Supplementary Fig. S2 — Inter-type, site-specific amino acid residue entropy. Residue variation estimated using Shannon entropy, wherein a value of zero reflects site-specific conservation and higher values indicate increasing degrees of site-specific variation. The positions of major structural elements are indicated. [file mmc2.pdf]

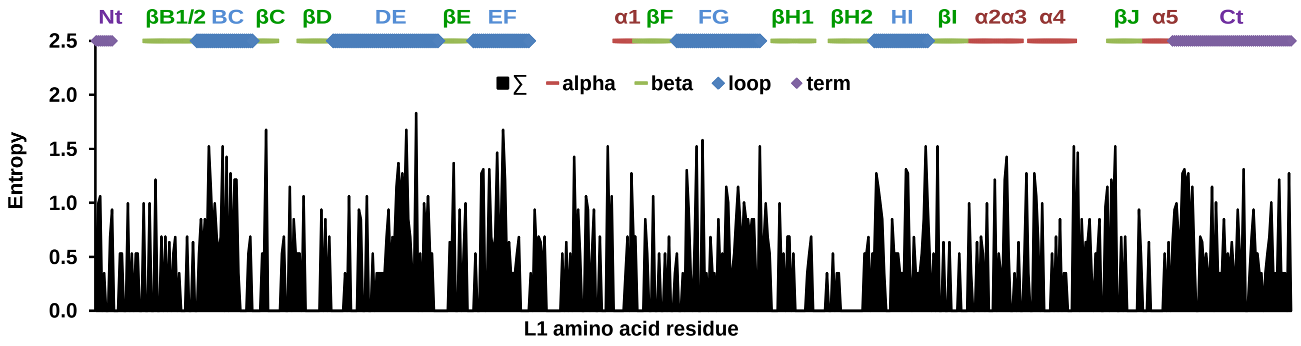

Supplementary Figure S2. Inter-type, site-specific amino acid residue entropy. Residue variation estimated using Shannon entropy, wherein a value of zero reflects site-specific conservation and higher values indicate increasing degrees of site-specific variation. The positions of major structural elements are indicated.
