# Supplementary material for: Amino acid sequence diversity of the major human papillomavirus capsid protein: Implications for current and next generation vaccines
Source: Infect Genet Evol. 2013 Aug;18:151–9. doi: 10.1016/j.meegid.2013.05.013 (PMC3769806; doi:10.1016/j.meegid.2013.05.013)
Supplement: Supplementary Table S1 — Accession numbers and source references. [file mmc3.pdf]

Supplementary Table S1. Accession numbers and source references

HPV	Source	Accession number(s)	Reference
6	China	AF335604	Zhang, M *et al.,* (2000)
	Slovenia	FM876121 - FM876165	Kocjan, BJ *et al.,* (2009)
		FM897134 - FM897165	
	Slovenia	FR751320 - FR751338	Kocjan, BJ *et al.,* (2011)
	England	AF067036 - AF067051	Caparros-Wanderley, W *et al.,* (1999)
	USA	AF092932	Kovelman, R *et al.,* (1999)
	China	AY015006 - AY015008	Direct Submission (2000)
	Slovenia	HE599226 - HE962032	Direct Submission (2012)
	Sweden	JN252314 - JN252323	Ure, AE *et al.,* (2012)
	USA	L41216	Hofmann, KJ *et al.,* (1995)
11	Slovenia	FN870688 - FN870750	Maver, PJ *et al.,* (2011)
	China	AF335602 - AF335603	Zhang, M *et al.,* (2000)
	China	EU918768	Wu, X *et al.,* (2009)
	Hungary	FR872717	Gall, T *et al.,* (2011)
	Hungary	HE574701 - HE574705	Direct Submission (2011)
	Slovenia	HE611258 - HE611274	Kocjan, BJ *et al.,* (2013)
		HE962365 - HE962368	
		HE962023 - HE962025	
	USA	JN644141 - JN644142	Direct Submission (2011)
	Thailand	JQ773408 - JQ773412	Chansaenroj, J *et al.,* (2012)
16	China	JX313693 - JX313723	Direct Submission (2012)
	Canada	GQ465877 - GQ479011	Cornut, G *et al.,* (2010)
	China	EF547252	Ma, ZH *et al.,* (2004)
	USA	U37217	Icenogle, JP *et al.,* (1995)
	USA	AF134175 - AF134178	White, WI *et al.,* (1999)
	China	AF084952, AF140365	Direct Submission (1999)
	China	AF393502	Gu, H *et al.,* (1999)
	Senegal	AF043286	Touze, A *et al.,* (1998)
	Philippines	AF043287	
	South Africa	AY177679	Varsani, A *et al.,* (2003)
	China	EU430672, EU430680, EU430688	Direct Submission (2008)
	India	DQ155283	Direct Submission (2005)
	Thailand	JQ004092 - JQ004099	Direct Submission (2011)
	Various	HQ644297 - HQ644299	Smith, B *et al.,* (2011)
	Slovenia	JQ067944, JN565303, JQ067943,	Sabol, I *et al.,* (2012)
		JN565302	
	China	EU918764	Wu, X *et al.,* (2009)
	East Asia	AF534061	Direct Submission (2002)
	China	FJ006723	Direct Submission (2008)
	USA	U89348	Seedorf, K *et al.,* (1985)
	Brazil	HM057182	Direct Submission (2010)
	Costa Rica	AF402678, AY686579 - AY686584	Chen, Z *et al.,* (2005)
	USA	AF534061, AF472508 - AF472509	Chen, Z *et al.,* (2005)
		AF536179 - AF536180	
	Thailand	FJ610147-FJ610152	Lurchachaiwong, W *et al.,* (2009)
	USA	AF125673	Flores, ER *et al.,* (1999)
	Germany	EU118173	Kirnbauer, R *et al.,* (1993)
31	Canada	Not submitted	Cornut, G *et al.,* (2010)
	Various	HQ537666 - HQ537687	Chen, Z *et al.,* (2011)
	USA	U37410	Icenogle, JP *et al.,* (1995)
33	China	EU918766	Wu, X *et al.,* (2009)
	Various	HQ537688 - HQ537707	Chen, Z *et al.,* (2011)
	Canada	GQ479012 - GQ479019	Cornut, G *et al.,* (2010)
52	China	GQ472848	Wu, XL *et al.,* (2010)
	Canada	EU077193 - EU077226	Gagnon, S *et al.,* (2007)
	China	GQ472848	Wu, XL *et al.,* (2010)
	Various	HQ537731 - HQ537751	Chen, Z *et al.,* (2011)
	China	JN874416 - JN874436	Chen, Q *et al.,* (2012)
58	China	EU918765	Wu, X *et al.,* (2009)
	China	FJ385261 - FJ385268	Wu, EQ *et al.,* (2009)

		FJ407192 - FJ407216	
	China	GQ472850	Wu, XL *et al.,* (2010)
	Various	HQ537752 - HQ537777	Chen, Z *et al.,* (2011)
	Various	HM639317 - HM639717	Chan, PK *et al.,* (2011)
	China	JX313752 - JX313772	Direct Submission (2012)
18	Costa Rica	EF202156 - EF202167	Chen, Z *et al.,* (2009)
	India	FJ528600	Direct Submission (2008)
	Thailand	GQ180784 - GQ180792	Lurchachaiwong, W *et al.,* (2010)
45	Costa Rica	EF202156- EF202167	Chen, Z *et al.,* (2009)
	USA	DQ080002	Buck, CB *et al.,* (2006)

References

Buck CB *et al.*; *PLoS Pathog* **2:**e69 (2006)
Caparros-Wanderley W *et al.*; *J Gen Virol* **80:**1025-33 (1999)
Chan PK *et al.*; *J Infect Dis* **203:**1565-73 (2011)
Chansaenroj J *et al.*; *Asian Pac J Cancer Prev* **13:**2619-23 (2012)
Chen Q *et al.*; *Int J Mol Med* **30:**535-44 (2012)
Chen Z *et al.*; *J Virol* **79:**7014-23 (2005)
Chen Z *et al.*; *J Virol* **83:**1443-55 (2009)
Chen Z *et al.*; *PLoS One* **6:**e20183 (2011)
Cornut G *et al.*; *J Med Virol* **82:**1168-78 (2010)
Flores ER *et al.*; *Virology* **262:**344-54 (1999)
Gagnon S *et al.*; *J Acquir Immune Defic Syndr* **44:**61-5 (2007)
Gall T *et al.*; *Antiviral Res* **92:**356-8 (2011)
Gu H *et al.*; *Zhonghua Shi Yan He Lin Chuang Bing Du Xue Za Zhi* **13:**17-9 (1999)
Hofmann KJ *et al.*; *Virology* **209:**506-18 (1995)
Icenogle JP *et al.*; *Virology* **214:**664-9 (1995)
Kirnbauer R *et al.*; *J Virol* **67:**6929-36 (1993)
Kocjan BJ *et al.*; *Virology* **391:**274-83 (2009)
Kocjan BJ *et al.*; *Infect Genet Evol* **11:**1805-10 (2011)
Kocjan BJ *et al.*; *J Infect Dis* **207:**583-7 (2013)
Kovelman R *et al.*; *J Gen Virol* **80 (Pt 9):**2445-51 (1999)
Lurchachaiwong W *et al.*; *Virus Genes* **39:**30-8 (2009)
Lurchachaiwong W *et al.*; *Intervirology* **53:**161-6 (2010)
Ma ZH *et al.*; *Zhonghua Yi Xue Za Zhi* **84:**987-91 (2004)
Maver PJ *et al.*; *J Med Virol* **83:**461-70 (2011)
Sabol I *et al.*; *PLoS One* **7:**e41045 (2012)
Seedorf K *et al.*; *Virology* **145:**181-5 (1985)
Smith B *et al.*; *PLoS One* **6:**e21375 (2011)
Touze A *et al.*; *J Clin Microbiol* **36:**2046-51 (1998)
Ure AE *et al.*; *J Virol* **86:**13790-4 (2012)
Varsani A *et al.*; *J Virol* **77:**8386-93 (2003)
White WI *et al.*; *J Virol* **73:**4882-9 (1999)
Wu EQ *et al.*; *J Gen Virol* **90:**1229-37 (2009)
Wu X *et al.*; *J Med Virol* **81:**693-702 (2009)
Wu XL *et al.*; *Virol Sin* **25:**8-17 (2010)
Zhang M *et al.*; *Zhongguo Yi Xue Ke Xue Yuan Xue Bao* **22:**463-6 (2000)
